# Antibacterial metal nanoclusters

**DOI:** 10.1186/s12951-022-01538-y

**Published:** 2022-07-16

**Authors:** Youkun Zheng, Min Wei, Haibin Wu, Fangyuan Li, Daishun Ling

**Affiliations:** 1 Key Laboratory of Medical Electrophysiology, Ministry of Education, Institute of Cardiovascular Research of Southwest Medical University, 646000 Luzhou, China; 2 Institute of Pharmaceutics, College of Pharmaceutical Sciences, Zhejiang University, 310058 Hangzhou, China; 3 Frontiers Science Center for Transformative Molecules, School of Chemistry and Chemical Engineering, National Center for Translational Medicine, Shanghai Jiao Tong University, 200240 Shanghai, China

**Keywords:** Metal nanoclusters, Nanoantibiotics, Antibacterial mechanisms, Bacterial infections

## Abstract

Combating bacterial infections is one of the most important applications of nanomedicine. In the past two decades, significant efforts have been committed to tune physicochemical properties of nanomaterials for the development of various novel nanoantibiotics. Among which, metal nanoclusters (NCs) with well-defined ultrasmall size and adjustable surface chemistry are emerging as the next-generation high performance nanoantibiotics. Metal NCs can penetrate bacterial cell envelope more easily than conventional nanomaterials due to their ultrasmall size. Meanwhile, the abundant active sites of the metal NCs help to catalyze the bacterial intracellular biochemical processes, resulting in enhanced antibacterial properties. In this review, we discuss the recent developments in metal NCs as a new generation of antimicrobial agents. Based on a brief introduction to the characteristics of metal NCs, we highlight the general working mechanisms by which metal NCs combating the bacterial infections. We also emphasize central roles of core size, element composition, oxidation state, and surface chemistry of metal NCs in their antimicrobial efficacy. Finally, we present a perspective on the remaining challenges and future developments of metal NCs for antibacterial therapeutics.

**Graphical Abstract:**

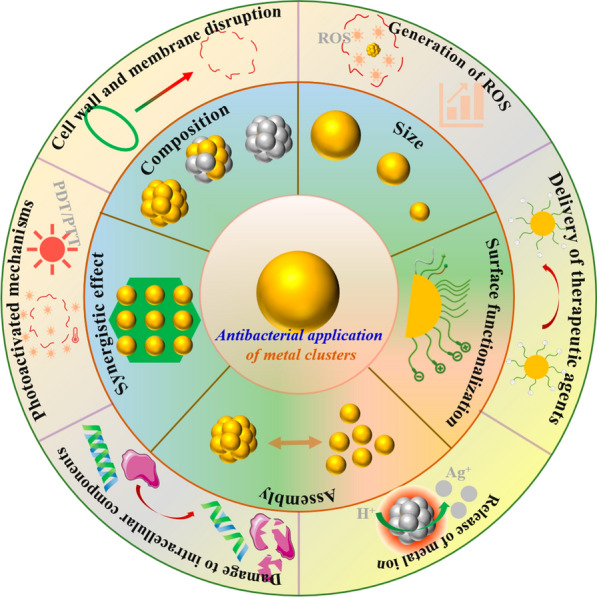

## Introduction

Bacterial infection is one of the greatest threats to global public health. In particular, the emergence of multidrug-resistant (MDR) superbugs makes conventional antibiotics ineffective, further exacerbating this threat [1]. According to the latest predictions, MDR bacterial infections will lead to 10 million annual deaths by 2050, more than those induced by cancer presently [2]. Faced with this serious challenge, new antibiotics have been developed to deal with the infamous superbug infections by chemically modifying existing antibiotics or exploiting new natural products [3–5]. Nevertheless, the development of new antibiotics is time-consuming and expensive, and the rapid resistance evolution of pathogenic bacteria can reduce or even inactivate the therapeutic activity of the most effective antibiotics [6, 7]. Besides, in order to eradicate MDR bacteria, high dosages or multiple of antibiotics may be required, which can induce serious adverse effects and uncertain outcomes [8]. Therefore, there is an urgent need to develop alternative antibacterial strategies, especially non-antibiotic agents, to combat the evolution of bacteria.

The discovery of the antimicrobial activity of nanomaterials, including metal-, metal oxide-, carbon-, quantum dot-, peptide- and polymer-based nanostructures, provides new opportunities to address the MDR crisis [9–13]. Compared with conventional antibiotics, antibacterial nanomaterials access novel antibacterial modalities against pathogens, which might not be attacked by their natural defense arsenal [14]. These rationally engineered nanomaterials kill pathogenic bacteria through diverse antibacterial mechanisms including cell wall and membrane destruction, oxidative damage, disruption of intracellular components, and the delivery of therapeutic agents [8]. For example, polymeric nanomaterials can be imparted with positive charge for interacting with anionic bacterial cell envelopes to perform antibacterial effect [15]. Graphene oxide nanoflakes exhibit antibacterial activity due to their sharp edges induced oxidative stress and photothermal activity [16]. Silver-carbon nanoparticles (NPs) selectively damage the membrane of gram-positive bacteria but keep safe to the membrane of germ cells [17]. AgFeO_2_ NPs perform both excellent antibacterial activity and magnetic response for targeted therapy and separation [18]. Moreover, these multiple antibacterial mechanisms can be designed to play a synergistic role in combating the MDR superbugs [13]. In view of the close interrelation between antibacterial effects and nanostructure, developing nanomaterials with highly controllable structure harbors the potential to construct novel effective antibacterial drugs according to the characteristics of pathogen infections.

**Scheme 1:**
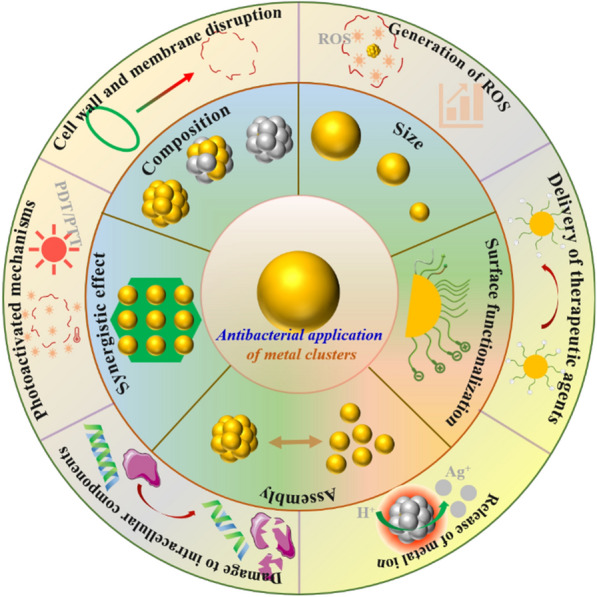
Metal NC-based nanoantibiotics for combating bacterial infections. Outer layer: six mechanisms of action of metal NCs on bacteria, including cell wall and membrane disruption, release of metal ion, generation of ROS, damage to intracellular components, damage to intracellular components, delivery of therapeutic agents, and photoactivated mechanisms. Inner layer: physiochemical factors affecting the antibacterial behavior of metal NCs, including size, composition, surface chemistry, assembly and synergistic effects with other antibacterial materials

Among the antibacterial nanomaterials, metal nanoclusters (NCs), the ultrasmall aggregates composed of a few to several hundred metal atoms with well-defined molecular structures [20], have attracted much attention in antibacterial application. The inherent advantages in structures (such as large surface area, precise size, morphology control, ease of surface modification) and physiochemical properties (such as unique optical, electromagnetic, and catalytic properties) enable metal NCs with precisely tunable antibacterial activity [7, 19]. For example, conventional AuNPs are inert for bacteria, while the potent antibacterial activity is emerged once decreasing their size to the nanocluster (NC) dimension (≤ 2 nm) [24]. Notably, the molecular-like properties of metal NCs are desirable to understand the antibacterial mechanisms of the nanostructures [21–23]. Moreover, the atomic-level manipulating and facilely tailoring of metal NCs empower them to act as multifunctional theranostic agents for photoluminescence guided bacterial infection therapy [25, 26]. The synthesis and physicochemical properties of metal NCs have been extensively discussed in previous reviews [27, 28]. In the present review, we focus on the research progress of metal NCs as a new generation of nanoantibiotics for biomedical applications (Scheme 1). We first give a brief introduction to the characteristics and possible advantages of metal NCs as nanoantibiotics, then we summarize the antimicrobial mechanisms of metal NCs, including cell wall and membrane disruption, release of metal ions, generation of reactive oxygen species (ROS), damage to intracellular components, delivery of antibacterial agents, and photoactivated mechanisms. Whereafter, we offer a comprehensive overview of the tailoring of physiochemical factors affecting the antibacterial behavior, such as the core size, element composition, and surface chemistry of metal NCs. The precise control of the properties of metal NC-based nanoantibiotics offers an in-depth insight of their antimicrobial mechanism, facilitating the rational design of next-generation antibacterial agents. Finally, a brief discussion of current challenges and future developments of metal NC-based nanoantibiotics is presented.

## Characteristics of metal NCs

Metal NCs have been considered as a new class of molecular-like aggregates consist of a few to several hundred metal atoms, whose sizes are comparable to the Fermi wavelength of electrons [20]. At this scale, the strong quantum effect of electrons causes continuous energy states to be divided into discrete electronic states [28]. As a result, metal NCs exhibit distinctive physicochemical properties, including significant Stokes shift, strong photoluminescence, good biocompatibility and HOMO-LUMO transition. As the missing link between single metal atoms and plasmonic metal NPs, metal NCs have received increasing attention in many fields, including antibacterial therapy as described in the following sections.

According to their fundamental compositions, the metal NCs for antibacterial applications can be roughly divided into AuNCs, AgNCs, CuNCs, alloy NCs and related composite nanostructures (Table 1). Among which, AgNCs and CuNCs usually possess outstanding antibacterial behaviors since the silver and copper elements have inherent broad-spectrum antibacterial activities [22, 29]. Nevertheless, the superior antibacterial effectiveness of NCs is not always accompanied with desirable biocompatibility in the mammalian cells, raising safety concerns for their clinical application [30]. In this regard, the “noblest” of metals, gold, has a greater advantage over silver and copper due to its biological inertia and high stability. Au-based nanostructures have also been extensively proven to possess excellent biocompatibility in living systems [31, 32], and their biocompatibility remains good even if the size is further reduced to the nanocluster range [33]. On the other hand, the antibacterial activities of Au-based agents usually need to be strengthened to reach the therapeutic goal through the rational regulation of their size, composition, and surface chemistry, as well as the incorporation of other antibacterial agents. Compared with single-metal NCs, metal alloy NCs (such as AuAg NCs and AuPt NCs) generally possess higher stability and tunable biological activities, and were widely applied in biomedical fields [34]. Metal alloy NCs with excellent antibacterial properties have also been recently developed [35]. In addition, metal NCs can be integrated with other therapeutic agents or functional materials, such as conventional antibiotics, polymers, and 2D nanomaterials, to realize synergistically enhanced antimicrobial effects.

**Table 1:** Antibacterial applications of metal NCs

Formulations	Target pathogens	Antibacterial mechanisms	References
Au_25_(MHA)_18_	Gram-negative bacteria; Gram-positive bacteria	Membrane damage; ROS generation; metabolic imbalance	[24]
GSH-Ag^+^-R NCs	Gram-negative bacteria; Gram-positive bacteria	Membrane damage; Ag^+^ release; ROS generation	[22]
AuDAMP	Gram-negative bacteria; Gram-positive bacteria	Membrane damage; ROS generation; DNA damage	[38]
Antimicrobial peptide-AuNCs	Gram-negative bacteria; Gram-positive bacteria	Membrane damage; delivery of antimicrobial peptide	[41]
QA-AuNCs	MRSA	Membrane damage; ROS generation; metabolic disturbance	[42]
MUTAB-AuNCs	Gram-negative bacteria; Gram-positive bacteria	Membrane damage; ROS generation	[43]
AuMS	Gram-negative bacteria; Gram-positive bacteria	Membrane damage	[44]
MSA-AgNCs	Gram-negative bacteria; Gram-positive bacteria	Ag^+^ release	[51]
TA-CuNCs	Gram-positive bacteria	Membrane damage; Cu^+^ release	[52]
Cys-AuNCs	*E. coli*	ROS generation	[57]
N-heterocyclic carbene-AuNCs	Gram-negative bacteria; Gram-positive bacteria; fungi	Membrane damage; ROS generation	[58]
AuPt bimetallic NCs	Gram-negative bacteria	Membrane damage; metabolic disturbance	[62]
Ag_3_NCs	*E. coli*	ROS generation; DNA damage	[63]
Histidine-AuNCs	Gram-negative bacteria	ROS generation; metabolic disturbance	[64]
Imidazole-AuNCs	*S. aureus*	ROS generation; metabolic disturbance	[65]
TPPMS/Ac_4_GlcSH-AuNCs	Gram-negative bacteria; Gram-positive bacteria	ROS generation; metabolic disturbance; DNA damage	[66]
Lys-AuNCs-Amp	MRSA and its persister	Delivery of ampicillin	[69]
Vancomycin-loaded Pep-AuNCs	*S. aureus*	Delivery of vancomycin	[70]
DNase-AuNCs	*E. coli; S. aureus*	PTT; PDT	[73]
Au_25_(Cys)_18_/crystal violet	*E. coli; S. aureus*	PDT	[75]
Chitosan-AgNCs	*E. coli*	PTT	[76]
Au_x_Ag_25−x_(MHA)_18_ alloy NCs	*S. aureus*	ROS generation	[35]
DNA-stabilized AgNCs	Gram-negative bacteria; Gram-positive bacteria	ROS generation	[86]
rAgNAs	MRSA	Membrane damage; Ag^+^ release	[91]
Dap-AuNCs	MRSA	Membrane damage; ROS generation; DNA damage	[95]
Dap-AgNCs	Gram-positive bacteria	Membrane damage; ROS generation; DNA damage	[96]
SFT/DT-AuNCs	Gram-negative bacteria; Gram-positive bacteria	Membrane damage	[97]
Dpep-AgNCs	Gram-negative bacteria	Cell wall damage; Ag^+^ release; delivery of antimicrobial peptide	[98]
AuNCs/Ho-GO nanosheets	Gram-negative bacteria; Gram-positive bacteria	Membrane damage; ROS generation; metabolic disturbance	[101]
Au_25_(MBA)_18_/MXene	*S. aureus*	Membrane damage; ROS generation; metabolic disturbance; DNA damage	[102]
Au NCs/CS	Gram-negative bacteria; Gram-positive bacteria	Membrane damage	[105]
Prot/MTU-AuNCs	*E. coli; S. aureus*	ROS generation	[108]
MSNs-AgNCs	Gram-negative bacteria; Gram-positive bacteria	Membrane damage; Ag^+^ release; ROS generation	[110]
pMBA-AuNCs	ESBL *E. coli*; MRSA	Membrane damage; intracellular component destruction	[111]
ABA-AuNCs	Gram-negative bacteria	Cell wall damage	[113]
QA-AuNCs/indocyanine green	MRSA	Membrane damage; PTT; PDT	[114]

**Scheme 2:**
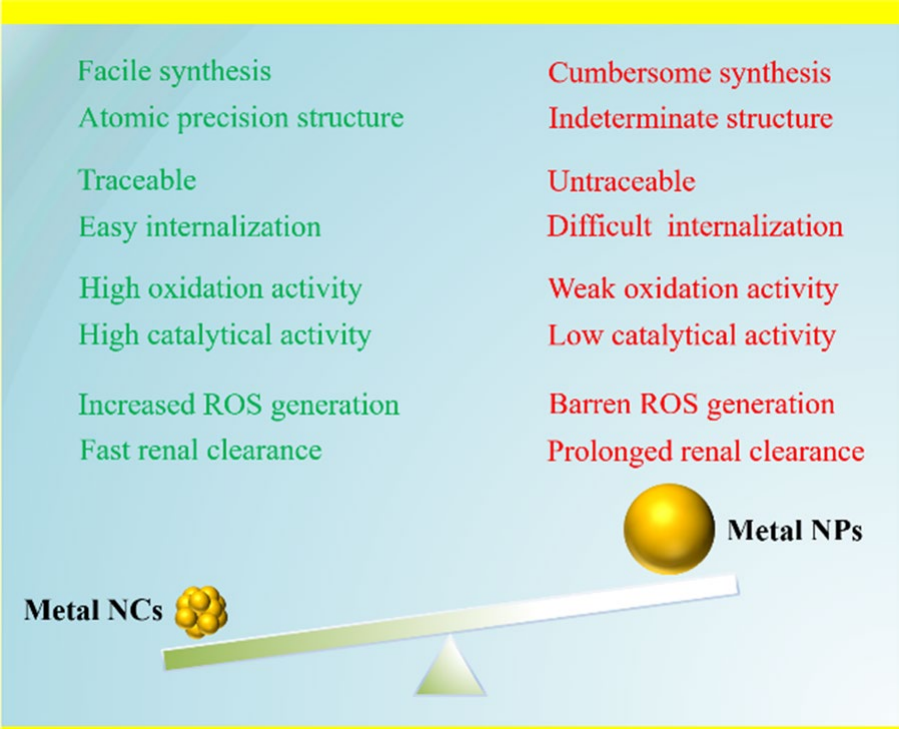
The unique advantages of metal NCs as antibacterial agents compared to plasmonic metal NPs

Compared with plasmonic metal NPs, metal NCs have several advantageous properties for antibacterial applications (Scheme 2). First, the metal clusters with atomic precision can be obtained via facile one-pot method. The atomic-precision structure endows a deep understanding of the structure-activity relationships of metal NC-based antibacterial therapy. In contrast, the synthesis of plasmonic metal NPs with specific and uniform morphological features is cumbersome and the resulting products are often heterogeneous, which greatly limits the understanding of their mechanisms of action. Moreover, due to the ultrasmall sizes, metal NCs can easily internalized into bacterial cells by traversing the cell wall pores, which greatly promotes their bactericidal activity by inducing ROS generation to oxidize bacterial membrane and disturb bacterial metabolism [36]. Meanwhile, owing to abundant active sites, ultrasmall metal NCs usually exhibit higher catalytic activity than metal NPs and thus induce higher levels of ROS generation, endowing metal NCs with stronger antibacterial activity [37, 38]. Moreover, the excellent photoluminescence properties make metal NCs traceable antimicrobials, which are rarely achieved by conventional metal NPs. Furthermore, the superior pharmacokinetics, biodegradation characteristics, and renal clearance of metal NCs are also crucial advantages for their clinical translation [39]. Benefiting from these advantages, ultrasmall metal NCs show significant promise as a new generation of nanoantibiotics for combating bacterial infections.

## Antibacterial mechanisms of metal NC-based nanoantibiotics

The ultrasmall size and diverse surface chemistry of metal NCs offer unique advantages for targeting pathogenic bacteria [40]. Metal NCs exhibit multiple antibacterial mechanisms, including cell envelope (cell wall and membrane) damage, release of metal ion, generation of toxic ROS, intracellular component destruction, delivery of antibacterial agents and photoactivated mechanisms. Table 1 presents the reported representative antibacterial metal NCs and their working mechanism. The antibacterial mechanisms mainly arise from the unique physicochemical properties of NCs, particularly, the multivalent interactions between NCs and bacteria via electrostatic attractions, hydrophobic interactions, Van der Waals forces, and receptor-ligand interactions [8]. In this section, we will discuss the antibacterial properties of metal NCs according to different antibacterial mechanisms.

### Cell wall and membrane disruption

The bacterial cell envelope is the first physical barrier to prevent antibacterial agents from entering the cell. The presence of teichoic acids (gram-positive bacteria) and lipopolysaccharide (gram-negative bacteria) on the cell wall render bacterial surfaces negatively charged, which promoting preferential electrostatic interactions with the positively charged nanomaterials [2]. Therefore, cationic nanostructures can readily bind to bacterial surface and interact with the cell envelope and damage the cell structure.

**Fig. 1:**
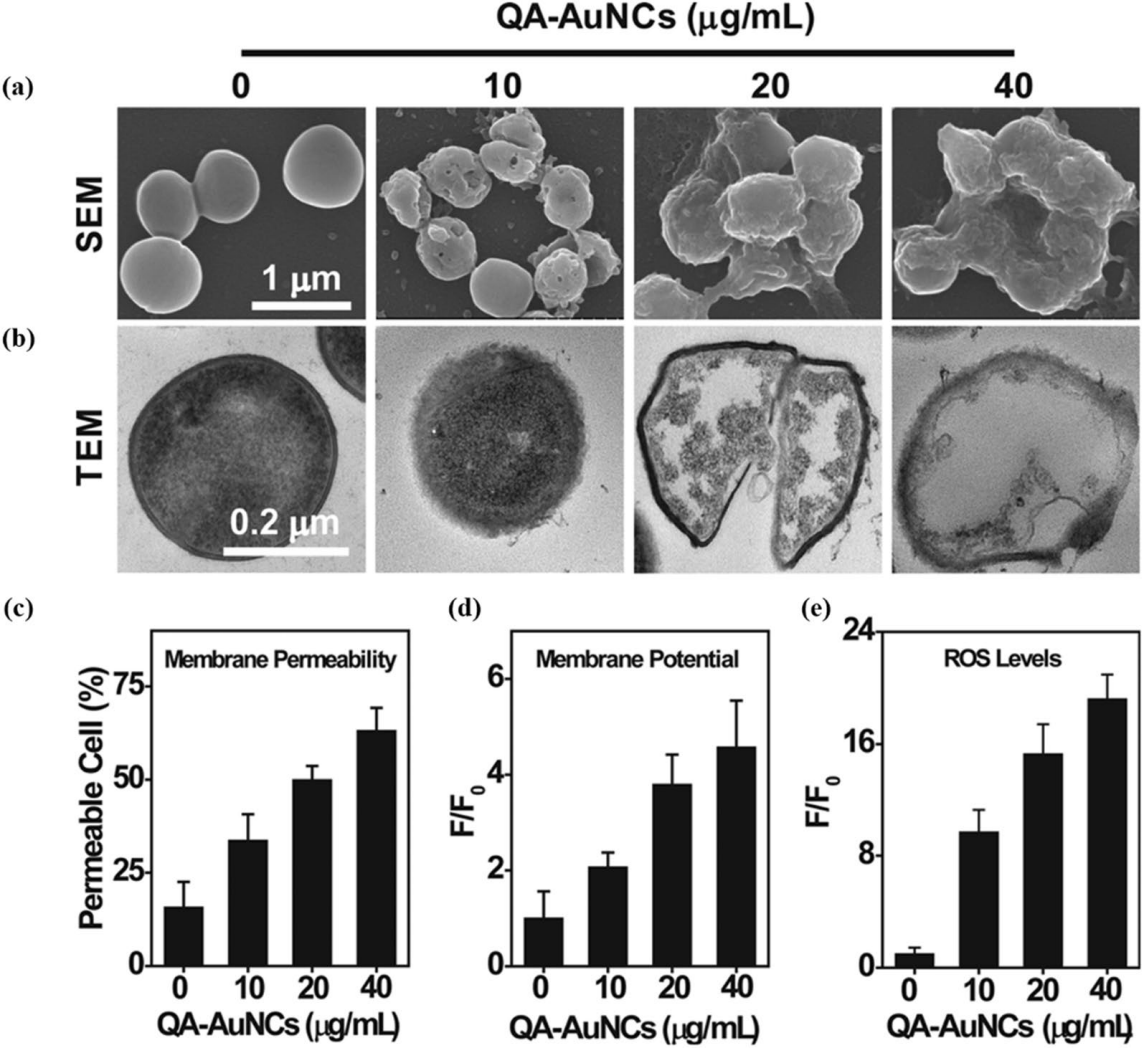
QA-AuNCs combat bacteria through a multipath mechanism. **a** SEM and **b** TEM images show the morphological changes of MRSA treated by QA-AuNCs. Increased membrane permeability (**c**), dissipation of the membrane potential (**d**) and high level of ROS (**e**) induced by QA-AuNCs (Reprinted with permission from [42]. Copyright (2018) Wiley-VCH Verlag & Co. KGaA, Weinheim)

A series of metal NC-based antibacterial strategies focus on targeting the negatively charged surface of bacteria [41, 42]. Xie et al. designed cationic AuNCs that functionalized with quaternary ammonium salts (QA-AuNCs), targeting methicillin-resistant *Staphylococcus aureus* (MRSA) [42]. The QA-AuNCs interact with bacterial cells via electrostatic interactions, leading to increased membrane permeability, dissipation of the membrane potential, and disruption of the membranes (Fig. 1a–d). The destruction of cell membrane integrity serves as the preliminary mechanism for their anti-MRSA activity. Our previous study also found that mercaptopyrimidine coated AuNCs (AuDAMP) contribute to the initiatory antibacterial mechanism by interacting with cell membranes [38]. Indeed, compared with conventional antibiotics, cationic AuNCs can bind to bacterial surfaces more firmly, which is the basis for the highly efficient antibacterial activities [43]. Moreover, Boda et al. found that the cell division and cell wall thickness of staphylococci treated with cationic AuNCs were significantly reduced, indicating that the biosynthesis of cell wall and membrane was inhibited [44]. Genechip microarray analysis revealed that the genes *Alt* and *SA1898* (encoding autolysin) related to bacterial membrane integrity were significantly up-regulated following the treatment with AuNCs [24]. RNA sequencing results also demonstrated that the expression of genes related to cell wall and membrane biosynthesis were significantly affected [45]. These results clearly showed that metal NCs can kill bacteria by inducing cell wall and/or membrane damage. In contrast to conventional antibiotics, the unique membrane disruption mechanisms of metal NCs can reduce the risk of emergence of bacterial resistance for long-term treatment [8, 46].

### Release of metal ion

For some metal NCs, especially AgNCs, the release of metal ions represents one of the decisive factors for their antibacterial activity, which is critical to the induction of cellular oxidative stress [7]. For instance, the production and release of silver ions (Ag^+^) is often triggered by the oxidation of Ag(0), which can in turn generate ROS and ultimately eliminate the bacteria [47]. It is well known that elevated ROS level can damage the proteins, enzymes and DNA in cells, thereby disrupting normal metabolism and function of the pathogenic bacteria [48]. In addition, the released metal ions can also directly bind to cellular components, such as amino acids, resulting in their dysfunction [49].

**Fig. 2:**
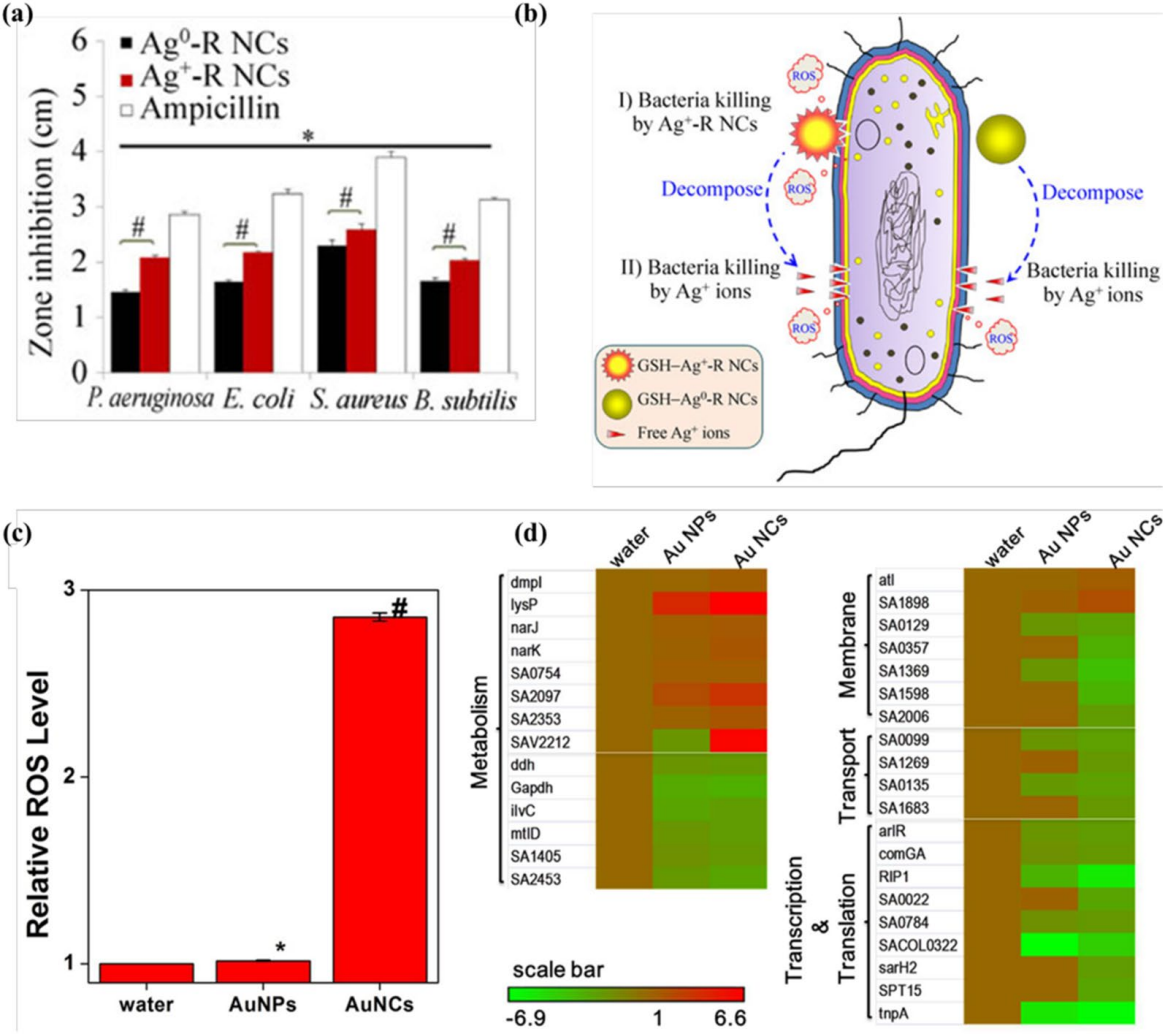
Metal NCs combat bacteria by releasing metal ions and inducing intracellular ROS generation. **a** Agar diffusion assay of zone inhibition by GSH-Ag^+^-R NCs, GSH-Ag^0^-R NCs, and ampicillin. **b** Schematic of possible antimicrobial mechanisms of GSH-Ag^+^-R NCs and GSH-Ag^0^-R NCs. Reprinted with permission from [22]. Copyright (2013) Tsinghua University Press and Springer-Verlag Berlin Heidelberg. **c** AuNCs induced intracellular ROS production. **d** Differential expression of genes related with cell metabolism, substrate transport, membrane integrity, and cell transcriptomic process were greatly affected following the AuNCs treatment. Red indicates gene up-regulation, and green indicates gene down-regulation (Reprinted with permission from [24]. Copyright (2017) American Chemical Society)

Compared with AgNPs, ultrasmall AgNCs have higher surface to volume ratio and are much more susceptible to oxidative dissolution, allowing a fast release of Ag^+^ for enhanced antibacterial performance. Yuan et al. found that the glutathione (GSH)-capped AgNCs exhibit potent antibacterial activity against *Pseudomonas aeruginosa*, which is attributed to the easily oxidized and released Ag^+^ on the surface of AgNCs [50]. Similarly, GSH-capped Ag^+^-rich AgNCs (GSH-Ag^+^-R NCs, with a predominance of Ag^+^ species on the surface of NCs) possess higher antibacterial activity than that of the GSH-Ag^0^-R NCs counterpart (Fig. 2a) [22]. The intact GSH-Ag^+^-R NCs have abundant local Ag^+^ on the surface are highly active in bacterial eliminating. Subsequently, a large amount of ROS will be generated to accelerate the dissociation of Ag^+^ from the NCs, and then initiate the second round of bacterial eliminating, further enhancing the antibacterial activity in a positive feedback loop (Fig. 2b). In contrast, the antibacterial activity of GSH-Ag^0^-R NCs is barely attributed to the dissociation of Ag^+^, which leads to a poor antibacterial activity. In a recent study, an AgNC-impregnated hydrogel with long-term and controlled release of Ag^+^ has been reported, which provides improved biofilm eradication capability [51]. In addition, the highly efficient antibacterial activity of Cu^+^ released-CuNCs has also been demonstrated [52].

### Generation of ROS

ROS is a general term describing the chemical substances formed upon incomplete reduction of oxygen, mainly including superoxide anion, hydrogen peroxide, singlet oxygen, and hydroxyl radical [48]. In living organisms, ROS play an important role in regulating various physiological functions of the entire life cycle [53]. However, the accumulation of excessive ROS leads to harmful oxidative stress, which can damage organisms via multiple working mechanisms, especially the consumption of intracellular vital reducing substances such as thiols in proteins [54].

Generation of ROS is one of the most essential antimicrobial mechanisms of metal NCs [55–58]. Besides the indirect ROS generation by metal ions, metal NCs can also directly catalyze the intracellular ROS in bacteria. Compared with large-sized AuNPs, ultrasmall AuNCs significantly up-regulate intracellular ROS level of bacteria (Fig. 2c) [24]. The excessive accumulation of ROS induced by AuNCs is responsible for the significant up-regulation of genes encoded the metabolic enzymes in the oxidative process (such as *dmpI* that encodes 4-oxalocrotonate tautomerase) and the down-regulation of antioxidant genes (such as *ilvC* that encodes ketol acid reductoisomerase and *Gapdh*) (Fig. 2d). Xie et al. further found that the generation of intracellular ROS by cationic QA-AuNCs plays an essential role in causing bacterial death (Fig. 1e) [42]. Furthermore, our study demonstrated that AuNCs-induced intracellular ROS generation mainly dependent on their inherent enzyme-mimic catalytic activity [38]. These AuNCs with inherent oxidase- and peroxidase-like catalytic capacities can up-regulate intracellular ROS levels, which make them promising candidates for next generation nanoantibiotics. Moreover, chemodynamic therapy (CDT), which is defined as the treatments through Fenton reaction or Fenton-like reaction mediated hydroxyl radical generation in acidic microenvironment, has emerged as a promising strategy for cancer and infection disease therapy [59–61]. Although the development of metal NCs based CDT agents is still in its infancy, considering the rich surface active sites of metal NCs to trigger efficient Fenton or Fenton-like reactions under the weak acidic conditions of biofilms, metal NCs-mediated CDT represents a potential alternative for the treatment of MDR bacterial infection.

### Damage to intracellular components

Homoeostasis of intracellular components and metabolic pathways are critical to the function and proliferation of bacterial cells. Several metal NCs have been found to eventually lead to bacterial death by interfering with these processes [38, 62–66]. These metal NCs-mediated damages include inhibition of ATP synthesis, consumption of reducing substances, reduction of enzyme activity and disruption of DNA. For example, atomically precise Au_25_NCs significantly reduce the metabolic activity and respiratory chain dehydrogenase activity of *Escherichia coli*, and destroy their DNA sequence structure [45]. RNA sequencing results indicated that the expression of bacterial genes related to glycolysis, oxidative phosphorylation, tricarboxylic acid cycle, and DNA replication repair were significantly disrupted by Au_25_NCs. By inducing the accumulation of intracellular ROS and disrupting the thiol-redox homeostasis of bacterial cells, the histidine-templated AuNCs show a potent therapeutic effect against ampicillin-resistant *E. coli* [64]. In another study, Zhao et al. found that the antibacterial mechanism of AuPt bimetallic NCs mainly involves dissipation of membrane potential and boosting intracellular ATP levels, which is not dependent on the ROS generation [62]. This unexpected mechanism of action may be resulted from the capability of AuPt bimetallic NCs to catalyze the generation of ATP and inhibit the process of ATP-consuming protein synthesis. In addition, Neissa et al. demonstrated that the bactericidal effect of Ag_3_ quantum clusters is attributed to the destruction of topoisomerase-DNA complexes, rather than the release of Ag^+^ [63]. Indeed, Ag_3_ quantum clusters can insert into DNA and induce prominent structural damage to the DNA [67]. The lifetime of these distortions was two to three orders of magnitude longer than that of conventional organic intercalants, such as proflavine and ethidium bromide, suggesting their excellent capability to damage bacterial DNA.

### Delivery of antibacterial agents

In addition to directly acting as therapeutic agents, metal NCs were also employed as nanocarriers for the delivery of existing antibacterial agents. Antibacterial agents can be loaded on the surface of metal NCs through covalent attachment or self-assembly [66–72]. The metal NCs-based delivery systems can broaden the antibacterial spectrum of conventional antibiotics and achieve controlled or targeted release of the drugs, thereby augmenting the therapeutic effect and reducing side effects [68]. For instance, an intelligent vancomycin delivery system was developed based on the custom-designed pentapeptide-mediated AuNCs [70]. The pentapeptide ligands contain a binding domain (D-alanine-D-alanine termini) with strong affinity for vancomycin. The self-assembled AuNCs complexes selectively release vancomycin in the presence of gram-positive bacteria due to the stronger binding affinity of vancomycin with bacterial cells than that with pentapeptide in AuNCs. The precise drug release profile of the AuNCs reduces the risk of systemic toxicity and potential side effects. In another study, an efficient antibiotic-AuNC hybrid system was developed by grafting ampicillin on the surface of lysozyme-templated AuNCs [69]. The antibacterial hybrids not only preserved the antibacterial activity against sensitive strains but also reversed the MRSA resistance towards ampicillin. In the presence of cis-2-decenoic acid, the hybrid systems can further inhibit MRSA persister, a hypopus of bacteria. This enhanced antibacterial performance of antibiotic-AuNC hybrid system is mainly attributed to the efficient inhibition of β-lactamase, multivalent binding to the bacterial surface and enhanced penetration.

### Photoactivated mechanisms

Phototherapy, including photodynamic therapy (PDT) and photothermal therapy (PTT), is promising in combating bacterial infection, since it can induce the photothermal effect or trigger the generation of ROS by employing the interaction between light and materials. Several non-antibacterial metal NCs can also exhibit potent antibacterial behavior through photoactivation mechanism. For example, DNase-functionalized AuNCs have been developed as photosensitizers to exhibit excellent photothermal and photodynamic properties under 808 nm laser radiation [73]. Highly efficient elimination of biofilm has been achieved owing to the synergistic PTT, PDT and enzymolysis effects of the DNase-functionalized AuNCs. In another study, Hwang et al. developed a rapid procedure to prepare high-quality Au_25_(Cys)_18_, which can kill pathogenic bacteria through photodynamic activity in the presence of crystal violet [74, 75]. Under low-flux white light radiation, non-antibacterial Au_25_(Cys)_18_ transfers photoelectrons to crystal violet to promote the redox reactions, thus resulting in enhanced ROS generation and bactericidal activity. In addition, chitosan-stabilized AgNCs have also been found to enhance the bactericidal ability through the PTT effect [76].

Overall, as a new generation of nanoantibiotics, metal NCs provide multiple antibacterial pathways to fight superbugs and circumvent antibiotic-resistant mechanisms. These working mechanisms are usually interrelated rather than acting individually. Appropriate tailoring of size, composition and surface performance provide a desirable avenue for the design of highly efficient novel antibacterial therapies.

## Physicochemical properties of metal NCs governing antibacterial properties

The physicochemical properties of nanostructures dominate their bioactivities and biomedical applications [77]. Therefore, investigating the nano-bio interface between metal NCs and bacteria is of great significance for a deep understanding of their antibacterial mechanisms and the design of potent antibacterial agents. Recent investigations have confirmed that the antibacterial effect of metal NC-based nanoantibiotics is highly correlated with their physicochemical properties, including size, composition, oxidation states, and surface chemistry [22, 24, 40, 62]. In this section, in order to gain deep insights into their structure–activity relationship, we will discuss how these physicochemical properties of metal NCs influence their antibacterial behaviors.

### Size

**Fig. 3:**
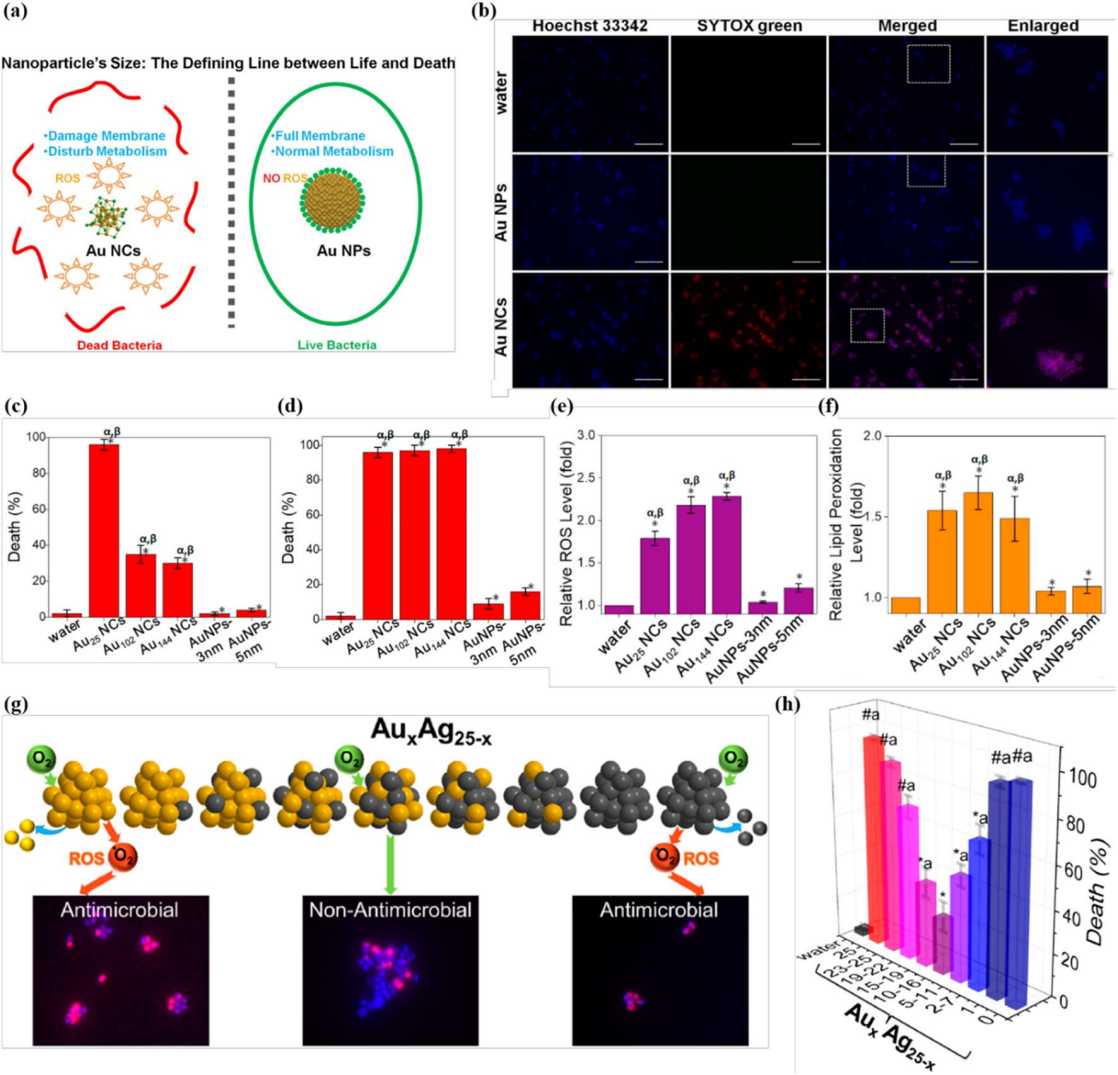
Size and composition affect the antibacterial activity of metal NCs. **a** Schematic illustration of the size regulation of AuNCs to significantly affect their antibacterial properties. **b** AuNCs showed high killing efficiency to *S. aureus*, which was absent when the cells were treated with AuNPs. The dead cells were visualized by SYTOX green (false color: red), whereas the Hoechst 33,342 (blue) helped to identify all cells. Scale bar: 25 μm. Reprinted with permission from [24]. Copyright (2017) American Chemical Society. Percentage of the dead *S. aureus* treated with AuNCs and AuNPs at the same Au atom concentration (**c)** and particle concentration (**d**) for 2 h. Relative intracellular ROS production level (**e**) and relative lipid peroxidation production level (**f**) of *S. aureus* after 2 h treatment of AuNCs and AuNPs at the same particle concentration. Reprinted with permission from [36]. Copyright (2020) KeAi Communications Co. Ltd. **g** Composition-dependent antimicrobial ability of full-spectrum Au_x_Ag_25−x_ alloy NCs. **h** Percentage of the dead *S. aureus* after treatment with Au_x_Ag_25−x_ alloy NCs (Reprinted with permission from [35]. Copyright (2020) American Chemical Society)

The size of metal-based NCs is critical to their antibacterial behaviors, as it significantly determines the processes of intracellular uptake, transport, accumulation and subsequent biological interactions. For instance, ultrasmall AuNCs can more easily enter bacterial cells and induce intracellular ROS burst, while large-sized AuNPs are incompetent even if they have consistent surface chemistry (Fig. 3a, b) [24]. In addition, reducing the size of AuNCs also greatly extended the antibacterial spectrum, since different sizes leading to divergent intracellular biochemical processes [78]. As the size of the AuNCs decreases, their capacities to interfere bacterial membrane permeability, membrane potential, and intracellular ROS and ATP levels are all significantly affected. In a detailed study, Zheng et al. set up a library of three different sized AuNCs of different gold atom numbers (Au_25_, Au_102_, and Au_144_) and two larger sized AuNPs (~ 3 and ~ 5 nm) capped by the same thiol ligand, *p*-mercaptobenzoic acid, and further investigate the size effect on antibacterial efficacy [36]. Au_25_, Au_102_, and Au_144_ can be easily internalized into the bacteria to achieve antibacterial activity. On the contrary, larger sized AuNPs exhibited ineffective internalization and no bactericidal effect was observed, indicating the vital role of size on antibacterial capability. Once the ultrasmall AuNCs have been internalized, they would work as a group to exhibit molecular-like antibacterial behavior, which displayed comparable antibacterial efficacy on the basis of the same molecular concentration of AuNCs (Fig. 3c, d). After internalization, the ultrasmall AuNCs can induce ROS generation to oxidize bacterial membrane and perturb cell metabolism (Fig. 3e, f), resulting in superior bactericidal effect. In addition, by adjusting the size of AgNCs, their antibacterial activity and cytotoxicity can be effectively balanced to produce biocompatible nano-antibacterial agents [79].

### Composition

Alloying is another promising strategy to construct metal NCs with enhanced physicochemical properties and improved antibacterial activities [80–83]. Compared with single metal composition, alloying also significantly influences the antibacterial behavior of metal NCs. For example, compared to non-antibacterial pure AuNCs and PtNCs, AuPt alloy NCs have superior antibacterial activity, which is governed by the composition ratio of gold and platinum atoms [62]. To understand the composition-dependent antibacterial behavior of metal NCs, a full-spectrum of alloy metal NCs, Au_*x*_Ag_25−*x*_(MHA)_18_ (MHA = 6-mercaptohexanoic acid) with *x* = 0 − 25, were developed and their antibacterial properties were investigated (Fig. 3g) [35]. As alloying enhances the stability of Au_*x*_Ag_25−*x*_(MHA)_18_, their ability to generate ROS is deactivated and thus affecting the antibacterial activity of NCs. Indeed, a U-shaped antibacterial profile was observed, where the alloyed NCs showed decreased antibacterial capability compared to AuNCs or AgNCs (Fig. 3h). This study showed that the composition of metal NCs finely regulates their antibacterial behavior, indicating the importance of a complete understanding of the composition-related properties and applications, which calls for multidisciplinary collaborative research.

### Surface chemistry

**Fig. 4:**
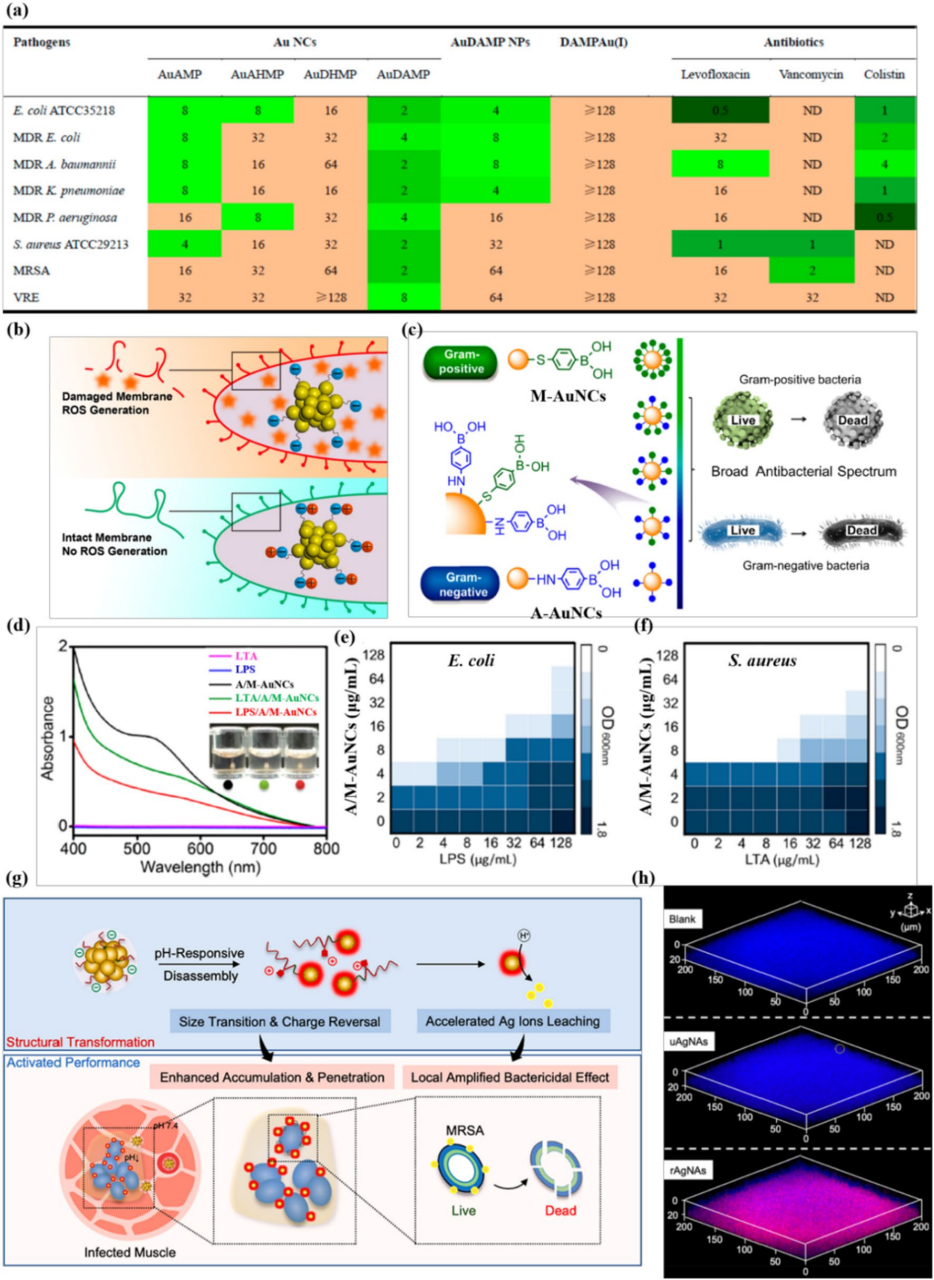
Surface chemistry modulates the antibacterial activity of metal NCs. **a** Comparison of antibacterial activities of mercaptopyrimidine analogues mediated AuNCs. Antibacterial activities indicated with MIC (µg/mL). AMP: 4-amino-2-mercaptopyrimidine; DAMP: 4,6-diamino-2-mercaptopyrimidine; AHMP: 4-amino-6-hydroxyl-2-mercaptopyrimidine; DHMP: 4,6-dihydroxyl-2-mercaptopyrimidine. Reprinted with permission from [38]. Copyright (2018) American Chemical Society. **b** Surface ligand chemistry of AuNCs determines their antimicrobial ability. Reprinted with permission from [40]. Copyright (2018) American Chemical Society. **c** Schematic illustration of the preparation process of phenylboronic acid-derivative-modified AuNCs by orchestrating the variation of ligands as tunable spectrum antibacterial agents. A/M-AuNCs exert bactericidal activity by interacting with LPS and LTA. **d** UV-vis spectra of the LPS, LTA, A/M-AuNCs, LPS/A/MAuNCs, and LTA/A/M-AuNCs. The antibacterial activity of A/M-AuNCs at different concentrations against *E. coli* or *S. aureus* in the presence of LPS and LTA at concentrations ranging from 0 to 128 µg/mL is plotted in parts **e** and **f**, respectively. Reprinted with permission from [88]. Copyright (2018) American Chemical Society. **g** Intelligent nanoantibiotics (rAgNAs), composed of ultrasmall AgNCs self-assembled with the assistance of acidity-responsive polymeric ligand, can accumulate in the biofilm with enhanced penetration, and can be selectively activated and rapidly release Ag^+^ in the acidic microenvironment of biofilm for improved therapeutic effect attributed to the acid-triggered disassembling of rAgNAs. **h** Enhanced accumulation and deep penetration of rAgNAs for the biofilm-amplified bactericidal effect (Reprinted with permission from [91]. Copyright (2019) American Chemical Society)

Surface modification is one of the most promising strategies for improving the biomedical applications of metal NCs. Through tailoring the surface chemistry, the antibacterial properties of metal NC-based nanoantibiotics can be easily regulated. In our previous report, the antibacterial activities of AuNCs can be modulated by surface ligand species [38]. Among the four investigated AuNCs modified by mercaptopyrimidine analogs with similar structures, the amino-rich ligands seem to endow the AuNCs stronger antibacterial activity and broader antibacterial spectrum (Fig. 4a). Notably, the amino-rich mercaptopyrimidine-AuNCs have also been further confirmed to be able to eliminate intracellular bacterial infections and concurrently regulate host cell immune responses [84]. Meng et al. found that grafting traditional herbal monomer cinnamaldehyde on the surface of histidine-mediated AuNCs (CA-AuNCs) can elevate ROS generation and concurrently deplete thiols in bacterial cells through ligand exchange, resulting in enhanced bacterial killing effect [85]. This work constructs oxidative stress amplifier (CA-AuNCs) through a ligand exchange strategy for combating MDR bacterial infections for the first time. Similarly, by simply tuning the oligonucleotide sequence, the physiochemical properties and antimicrobial performances of the DNA-templated AgNCs can be easily adjusted [86]. Further structural studies have shown that the AgNCs possess different structure and stability, which might be one of the key factors regulating their antibacterial activity. In addition, the influence of the spatial assembly of oligonucleotide sequences on the antibacterial behavior of DNA-scaffolded AgNCs was also demonstrated [87].

The surface charge of metal NCs is another important factor affecting their antibacterial behavior. It is generally believed that positively charged surface of antibacterial nanomaterials would favor close interaction with the negatively charged bacterial surface, resulting in improved antibacterial efficacy [2]. Indeed, a series of cationic metal NCs have been successfully developed as nanoantibiotics [41, 42]. By fine-tuning the surface charges of AuNCs, however, Zheng et al. proposed an antibacterial behavior in stark contrast to this paradigm [40]. They reported that ultrasmall AuNCs with more negative charges show better antimicrobial effects due to the induction of higher intracellular ROS levels (Fig. 4b). This surprising finding suggests the complexities of the NC-bacterial cell interactions and sheds some light on the design of high-performance metal NC-based nanoantibiotics.

A recent study has found that the ligand density of metal NCs can also contribute to different antibacterial behaviors [88]. By regulating the density of phenylboronic acid on surface of AuNCs by adjusting the ratio of different anchoring groups, thiol (-SH) or amine (-NH_2_) groups, an AuNCs with tunable antibacterial capability was synthesized (Fig. 4c). The AuNCs modified by mercaptophenylboronic acid (M-AuNCs) and aminophenylboronic acid (A-AuNCs) specifically bind to lipoteichoic acid (LTA) of gram-positive bacteria and lipopolysaccharide of gram-negative bacteria, respectively, resulting in potent and tunable antibacterial behavior (Fig. 4d-f). This adjustable antibacterial behavior is expected to be promising in personalized therapy.

Metal NCs can also be activated to combat bacterial biofilms through surface modulation [43, 51, 73]. The formation of biofilm is considered to be the key to antibiotic resistance, which serves as a natural barrier for antibiotic penetration and activation [89]. Conventional antibacterial therapeutics exhibit limited penetration and reduced activity in the acidic microenvironment (pH values of 4.5–6.5) of the bacterial biofilm [90]. To overcome this barrier, our group developed a pH-responsive biofilm elimination strategy through the self-assembly of ultrasmall AgNCs via customized pH-sensitive charge reversal ligands [91]. The surface-assembled nanoantibaiotics (rAgNAs) can selectively activate the antibacterial activity in the acidic biofilm microenvironment. Under non-acidic conditions, the antibacterial activity of rAgNAs is extinguished because the release of toxic Ag^+^ is inhibited by surface assembly (Fig. 4g). Once entering the acidic biofilm microenvironment, rAgNAs not only show charge reversal to promote local accumulation and retention but also disassemble into small AgNCs, thus enabling deep penetration and accelerated the Ag^+^ release for significantly enhanced antibacterial activity (Fig. 4 h). In addition, since the release of Ag^+^ is inhibited in the natural physiological environment, the damage of the AgNCs to mammalian cells is also effectively avoided. This biofilm-responsive nano-antibacterial strategy has shown great potential in the treatment of drug-resistant bacterial biofilm infections. Moreover, cationic thiolate modified AuNCs show highly efficient antibacterial effect against mature biofilm, likely due to the excellent permeability of positively charged AuNCs into biofilm [43]. DNase-functionalized AuNCs can hydrolyze DNA in extracellular polymeric substances matrix and induce oxidative stress with photoactivation to eradicate biofilm [73]. Overall, surface engineering represents a promising approach to enhance the antibacterial effect of metal NC-based nanoantibiotics.

Except for size, composition and surface chemistry, other physicochemical properties of metal NCs can also affect their antimicrobial effect. For instance, thiolated AuNCs with more loosely bound Au(I)-thiolate surface motifs (semi-rigid structure) have better antimicrobial activity was demonstrated [92]. In addition, it is reported that the oxidation states of Ag atoms in AgNCs are also critical on their antimicrobial effect, and AgNCs with higher Ag(I) content had a stronger killing effect [22]. In conclusion, by systematically investigating the influence of each factor on the antimicrobial capability and the underlying antibacterial mechanism, we can rationally design highly efficient antimicrobial metal NCs by tailoring their size, composition, surface chemistry, structure, and oxidation state.

## Synergistic antibacterial metal NCs

**Fig. 5:**
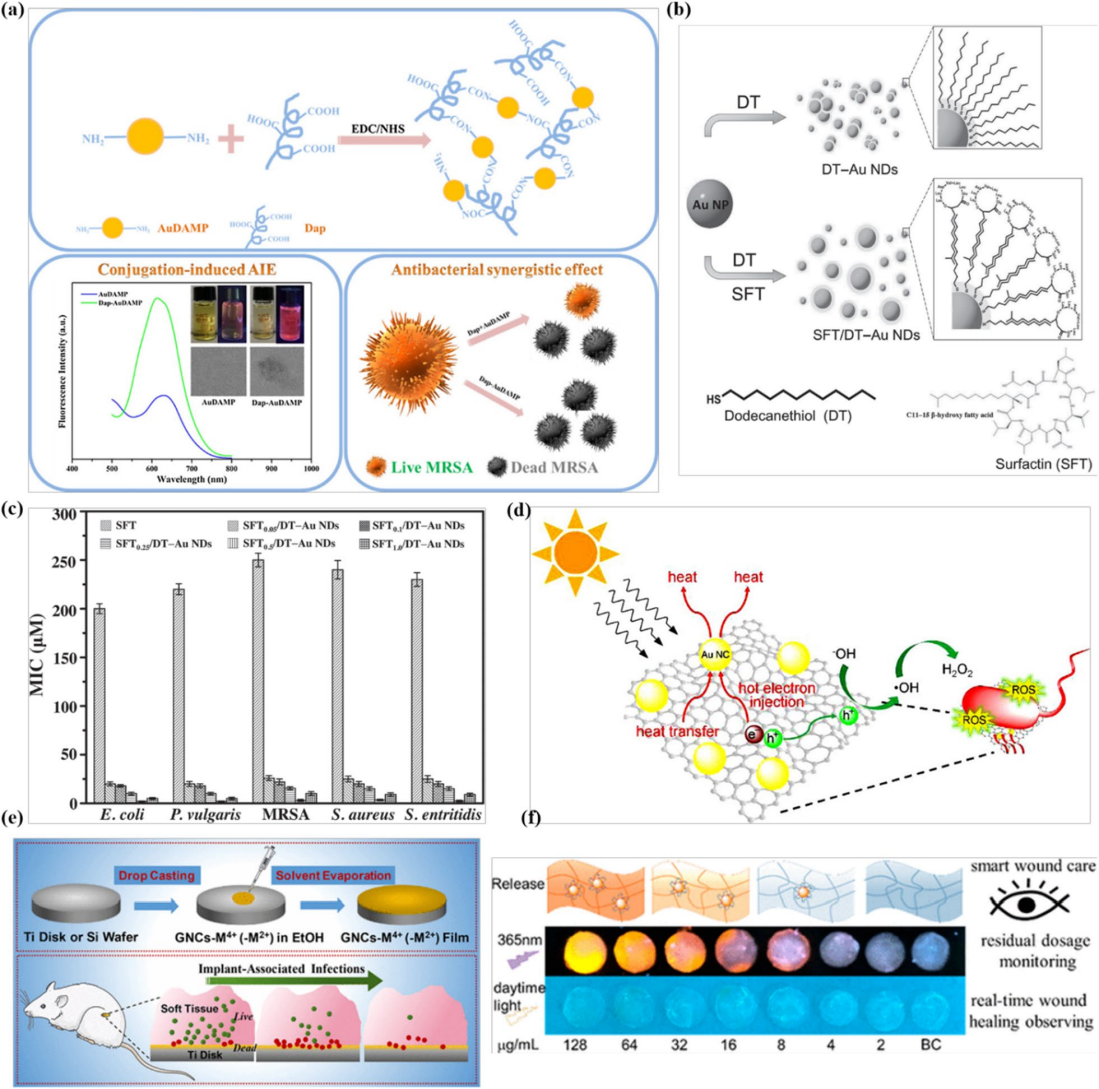
Synergistic antibacterial activity of metal NCs. **a** Schematic illustrations of the conjugation strategy for antibacterial AuNCs and daptomycin, conjugation-induced aggregation-induced emission enhancement, and antibacterial synergistic effect. Reprinted with permission from [95]. Copyright (2019) Elsevier. **b** Synthesis of photoluminescent SFT/DT-Au NDs. **c** Comparison of MICs (in terms of the concentration of SFT) of SFT, SFT_0.05_/DT-Au NDs, SFT_0.1_/DT-Au NDs, SFT_0.25_/DT-Au NDs, SFT_0.5_/DT-Au NDs, and SFT_1.0_/DT-Au NDs against *E. coli*, *P. vulgaris*, MRSA, *S. aureus*, and *S. enteritidis*, respectively. Reprinted with permission from [97]. Copyright (2015) Wiley-VCH Verlag & Co. KGaA, Weinheim. **d** Schematic illustration of the antibacterial mechanism of Au-GO nanosheets under visible light irradiation. Reprinted with permission from [100]. Copyright (2020) Elsevier. **e** An AuNC constructed mixed-metal metal-organic network film for combating implant-associated infections. Reprinted with permission from [111]. Copyright (2020) American Chemical Society. **f** Schematic illustration of the bacterial cellulose scaffold loaded with aminobenzeneboronic acid (ABA)-modified AuNCs as antibacterial wound dressing to address the issue of MDR-infected skin wounds (Reprinted with permission from [113]. Copyright (2021) American Chemical Society)

Metal NC-based nanoantibiotics can be integrated with other therapeutic or functional materials to realize synergistically enhanced antimicrobial effects. Combination of antibacterial therapies can attack bacteria from different fronts, which is one of the most common strategies for the treatment of severe MDR bacterial infections [93, 94]. The enhanced antibacterial activity of metal NCs can be realized by integrating NCs with other agents such as conventional antibiotics, polymers, and 2D nanomaterials to form a complementary hybrid. For instance, we recently demonstrated a synergistically enhanced antibacterial hybrid by conjugating amino-rich mercaptopyrimidine-AuNCs (AuDAMP) with a cyclic lipopeptide antimicrobial peptide daptomycin (Dap) (Fig. 5a) [83]. The prepared antibacterial hybrids (Dap-AuDAMP) integrate the antibacterial capabilities of both agents and render an enhanced synergistic effect. Relying on the daptomycin-induced disruption of bacterial membrane, the bacterial cell internalization of AuNCs is greatly enhanced. The internalized AuNCs can generate ROS continuously in bacterial cells and then induce bacterial death. The continuous ROS generation also limit the development of bacterial resistance. In addition, a novel aggregation-induced emission (AIE) pattern between the AuDAMP and daptomycin was also observed. Similar synergistic antibacterial effects can also be obtained by integration of antibacterial AgNCs with daptomycin [96]. Therefore, this universal concept can be further extended to other metal NCs and antimicrobial peptides. In another study, a self-assembly strategy of metal NCs and antimicrobial peptides was also developed [97]. Antimicrobial peptide surfactin (SFT) is bound to the surface of 1-dodecanethiol-capped AuNCs through non-covalent bonds to form a highly efficient antibacterial assembly (SFT/DT-AuNDs) (Fig. 5b). Compared with SFT, the SFT/DT-AuND assemblies show improved antimicrobial activity since they possess a lower minimum inhibitory concentration (> 80-fold) than that of SFT (Fig. 5c). In addition, synergistically enhanced antimicrobial behavior has also been successfully achieved by directly using antimicrobial peptides as surface ligands to synthesize metal NCs [98].

As emerging functional materials, 2D nanomaterials such as graphene oxide (GO) and MXene nanosheets, are also employed to synergistically enhance the antibacterial properties of metal NCs [99–103]. These 2D nanomaterials generally have distinctive antibacterial action mechanisms. For example, GO nanosheets can physically cut through bacterial membranes and induce ROS generation [104]. By decorating AuNCs into GO nanosheets, a highly efficient antibacterial nanohybrid was developed [100]. The assembled nanosheets can simultaneously produce massive heat and generate high levels of ROS to inactivate bacteria under visible light irradiation (Fig. 5d). In comparison with bare AuNCs and GO nanosheets, these GO-AuNC nanohybrids show an enhanced antimicrobial activity towards gram-positive and gram-negative bacteria. Later, Zheng et al. constructed a synergistic antibacterial hybrid by the integrating of antibacterial AuNCs and the paramagnetic holmium ions (Ho^3+^) onto GO nanosheets [101]. The complexed holmium ions can help the nanohybrids to be vertically aligned under weak magnetic fields, which offer a high-density sharp edge with preferential orientation to effectively pierce the bacterial membrane. Meanwhile, the integrated AuNCs can effectively internalize into bacterial cells to induce high levels of ROS, which strongly disturb the cell metabolism. These antibacterial nanohybrids employ both physical (by oriented GO nanosheets) and chemical (by integrated AuNCs and GO nanosheets) mechanisms to realize synergistic antibacterial performances. Similarly, by conjugating antibacterial AuNCs onto titanium carbide (MXene), the synergistic multi-mechanism antibacterial performance is also achieved [102].

Integration with natural polymers, such as chitosan (CS), can also lead to synergistically enhanced antibacterial activity of metal NC-based nanoantibiotics. For example, an efficient antibacterial nanoaggregate was developed based on the self-assembly of mercaptosuccinic acid (MSA)-protected AuNCs and chitosan [105]. These self-assembled nanoaggregates displayed enhanced antimicrobial activity against both gram-negative and gram-positive bacteria compared with individual components. A composite hydrogel that encapsulates ultrasmall AgNCs into chitosan matrixes to enhance antibacterial behavior and promote tissue reconstruction has also been reported, showing great translation potential [106, 107]. In addition, several synergistic antimicrobial strategies based on the assembly of metal NCs and other materials, such as recognition proteins, upconversion NPs, and mesoporous silica NPs, have also been established [108–110]. Overall, these studies provide new options for improving the antibacterial properties of metal NCs, especially in dealing with notorious superbugs’ infections.

In addition, there have been several reports on the use of metal NCs as effective ingredients in antibacterial practices. For instance, Chu et al. constructed an antibacterial film composed of AuNC-based mixed-metal metal-organic network on titanium disks to effectively inhibit implant-related infections (Fig. 5e) [111]. The generalizable modular procedure of the AuNC-metal-organic networks is amenable to accelerate the modification of metal surfaces for inhibiting implant-associated infections. Similarly, coating the cationic AuNCs on the orthodontic device (aligner) can effectively combat the formation of *Streptococcus mutans* biofilm [112]. The anti-biofilm activity of the coated AuNCs can be maintained for at least 3 months, even after repeated usage. In order to visually monitor nanotherapeutic-loaded wound dressings, a novel wound dressing by integrating the fluorescence of the nanotherapeutic and the transparency of the scaffold was developed [113]. During the bacteria-infected wound healing process, the fluorescence intensity of the therapeutic AuNCs in the transparent bacterial cellulose scaffolds decreases as the release of the nanotherapeutic into the wound, which indicates the replacement of the dressing when the residual concentration of the AuNCs is lower than the minimum inhibitory concentration (Fig. 5f). Therefore, by the real-time monitoring of the dressing state, wound damage caused by frequent dressing replacement can be avoided. Furthermore, this visible strategy can be extended to medical devices to realize high-precision real-time monitoring during their service life. Recently, Zhuo et al. prepared a nanoantibiotic that could cross the blood-brain barrier by combining QA-AuNCs and indocyanine green [114]. With the help of the near-infrared laser, the nanoantibiotics could effectively cross the blood-brain barrier and treat intracranial MRSA infection at low doses through a triple-combination synergistic therapy of direct-killing, PTT, and PDT. Compared with traditional vancomycin treatment, the synergistic treatment was significantly less toxic to the liver and kidney and thus would be a safe strategy for intracranial MRSA-infection therapy.

## Summary and perspective

As an innovative type of versatile nanomedicine, metal NCs have been recently found to possess attractive prospects in the treatment of increasingly serious MDR bacterial infections. In the present review, we provide a comprehensive review of the current status of ultrasmall metal NCs in antibacterial applications, including antibacterial mechanisms, structure-activity relationships, and synergistic effects. The general mechanisms of metal NC-based nanoantibiotics targeted bacterial infections include cell wall and/or membrane damage, metal ions release, intracellular ROS generation, destruction of intracellular components, the delivery of therapeutic agents, and photoactivated mechanisms. The physicochemical properties of metal NCs, including size, composition, oxidation state, and surface chemistry, govern their antibacterial behaviors. Metal NC-based nanoantibiotics can be integrated with other therapeutic or functional materials, such as antimicrobial peptides, 2D nanomaterials, and polymers, to form complementary nanohybrids with synergistically enhanced antibacterial effects. Based on the regulation of the physicochemical properties of the metal NCs and further functionalization, the multifarious personalized antibacterial nanomaterials can be fabricated for precision medicine.

However, there are several challenges remain to be addressed for further translation of these metal NC-based nanoantibiotics. First, although the antibacterial mechanisms of atomically precise metal NCs have been systematically investigated, the understanding of the dynamic nano-bio interaction is still insufficient, which is also an underexplored field [115]. We can take full advantage of the atomically precise physiochemical characterization of metal NCs to investigate the nano-bio interaction for advancing the rational design of nanoantibiotics. Second, given that the complex effect of alloying on the antimicrobial activity of metal NCs is extraordinary [35], it is suggested that the influence of this method on the antimicrobial behavior of metal NCs should be extensively investigated. Especially, alloying is also a promising strategy to improve the photoluminescence efficacy and structural stability of metal NCs, which is essential for their diverse biomedical applications such as traceable nanoantibiotics [62, 116]. Third, more attention should be paid to the effect of the chirality and isomerization of surface ligands on the antimicrobial activity of metal NCs, because these properties haven been reported to profoundly affect the biological interactions of nanomaterials [117–119]. Finally, although several barriers including lysosomes escaping, mitochondria targeting and mitochondria membrane penetration alleviate the toxicity of cationic nanostructures to mammalian cells [120], the in vitro and in vivo biosafety of the metal NC-based nanoantibiotics should be fully considered and evaluated to facilitate their clinical translation [121]. We believe that through the joint efforts of scientists in different fields such as nanobiotechnology, materials chemistry, pharmaceutical science and clinical medicine, antibacterial metal NCs, as effective next generation nanoantibiotics, hold a bright future for dealing with the serious crisis of MDR bacterial infections.

## Data Availability

Not applicable.

## Abbreviations

AHMP: 4-Amino-6-hydroxyl-2-mercaptopyrimidine
AIE: Aggregation-induced emission
AMP: 4-Amino-2-mercaptopyrimidine
CDT: Chemodynamic therapy
CS: Chitosan
DAMP: 4,6-Diamino-2-mercaptopyrimidine
DHMP: 4,6-Dihydroxyl-2-mercaptopyrimidine
DT: 1-Dodecanethiol
GO: Graphene oxide
GSH: Glutathione
LPS: Lipopolysaccharide
LTA: Lipoteichoic acid
MDR: Multidrug-resistant
MHA: 6-Mercaptohexanoic acid
MRSA: Methicillin-resistant *Staphylococcus aureus*
MSA: Mercaptosuccinic acid
NCs: Nanoclusters
NPs: Nanoparticles
QA: Quaternary ammonium salts
ROS: Reactive oxygen species
SEM: Scanning electron microscope
SFT: Surfactin
TEM: Transmission electron microscope

## References

[1] Blair JM, Webber MA, Baylay AJ, Ogbolu DO, Piddock LJ (2015). Molecular mechanisms of antibiotic resistance. Nat Rev Microbiol.

[2] Gupta A, Mumtaz S, Li CH, Hussain I, Rotello VM (2019). Combatting antibiotic-resistant bacteria using nanomaterials. Chem Soc Rev.

[3] Kim W, Zhu W, Hendricks GL, Tyne DV, Steele AD, Keohane CE, Fricke N, Conery AL, Shen S, Pan W, Lee K, Rajamuthiah R, Fuchs BB, Vlahovska PM, Wuest WM, Gilmore MS, Gao H, Ausubel FM, Mylonakis E (2018). A new class of synthetic retinoid antibiotics effective against bacterial persisters. Nature.

[4] Guan D, Chen F, Qiu Y, Jiang B, Gong L, Lan L, Huang W (2019). Sulfonium, an underestimated moiety for structural modification, alters antibacterial profile of vancomycin against multidrug-resistant bacteria. Angew Chem Int Ed.

[5] Mitcheltree MJ, Pisipati A, Syroegin EA, Silvestre KJ, Klepacki D, Mason JD, Terwilliger DW, Testolin G, Pote AR, Wu KJY, Ladley RP, Chatman K, Mankin AS, Polikanov YS, Myers AG (2021). A synthetic antibiotic class overcoming bacterial multidrug resistance. Nature.

[6] Huh AJ, Kwon YJ (2011). “Nanoantibiotics”: a new paradigm for treating infectious diseases using nanomaterials in the antibiotics resistant era. J Controlled Release.

[7] Zheng K, Setyawati MI, Leong DT, Xie J (2018). Antimicrobial silver nanomaterials. Coordin Chem Rev.

[8] Makabenta JMV, Nabawy A, Li CH, Schmidt-Malan S, Patel R, Rotello VM (2021). Nanomaterial-based therapeutics for antibiotic-resistant bacterial infections. Nat Rev Microbiol.

[9] Fang G, Li W, Shen X, Perez-Aguilar JM, Chong Y, Gao X, Chai Z, Chen C, Ge C, Zhou R (2018). Differential Pd-nanocrystal facets demonstrate distinct antibacterial activity against gram-positive and gram-negative bacteria. Nat Commun.

[10] Wang Z, Wang X, Wang Y, Zhu Y, Liu X, Zhou Q (2021). NanoZnO-modified titanium implants for enhanced anti-bacterial activity, osteogenesis and corrosion resistance. J Nanobiotechnol.

[11] Barros CHN, Hiebner DW, Fulaz S, Vitale S, Quinn L, Casey E (2021). Synthesis and self-assembly of curcumin-modified amphiphilic polymeric micelles with antibacterial activity. J Nanobiotechnol.

[12] Xu S, Chang L, Hu Y, Zhao X, Huang S, Chen Z, Ren X, Mei X (2021). Tea polyphenol modified, photothermal responsive and ROS generative black phosphorus quantum dots as nanoplatforms for promoting MRSA infected wounds healing in diabetic rats. J Nanobiotechnol.

[13] Gao W, Zhang L (2021). Nanomaterials arising amid antibiotic resistance. Nat Rev Microbiol.

[14] Linklater DP, Baulin VA, Juodkazis S, Crawford RJ, Stoodley P, Ivanova EP (2021). Mechano-bactericidal actions of nanostructured surfaces. Nat Rev Microbiol.

[15] Lam SJ, Wong EH, Boyer C, Qiao GG (2018). Antimicrobial polymeric nanoparticles. Prog Polym Sci.

[16] Khan MS, Abdelhamid HN, Wu HF (2015). Near infrared (NIR) laser mediated surface activation of graphene oxide nanoflakes for efficient antibacterial, antifungal and wound healing treatment. Colloids Surf B Biointerfaces.

[17] Yousef MS, Abdelhamid HN, Hidalgo M, Fathy R, Gómez-Gascón L, Dorado J (2021). Antimicrobial activity of silver-carbon nanoparticles on the bacterial flora of bull semen. Theriogenology.

[18] Abdelhamid HN, Talib A, Wu HF (2015). Facile synthesis of water soluble silver ferrite (AgFeO_2_) nanoparticles and their biological application as antibacterial agents. RSC Adv.

[19] Zheng Y, Jiang H, Wang X (2020). Facet-dependent antibacterial activity of Au nanocrystals. Chinese Chem Lett.

[20] Zhang L, Wang E (2014). Metal nanoclusters: new fluorescent probes for sensors and bioimaging. Nano Today.

[21] Tang M, Zhang J, Yang C, Zheng Y, Jiang H (2020). Gold nanoclusters for bacterial detection and infection therapy. Front Chem.

[22] Yuan X, Setyawati MI, Leong DT, Xie J (2014). Ultrasmall Ag^+^-rich nanoclusters as highly efficient nanoreservoirs for bacterial killing. Nano Res.

[23] Wang S, Wang Y, Peng Y, Yang X (2019). Exploring the antibacteria performance of multicolor Ag, Au, and Cu nanoclusters. ACS Appl Mater Interfaces.

[24] Zheng K, Setyawati MI, Leong DT, Xie J (2017). Antimicrobial gold nanoclusters. ACS Nano.

[25] Jin R, Zeng C, Zhou M, Chen Y (2016). Atomically precise colloidal metal nanoclusters and nanoparticles: fundamentals and opportunities. Chem Rev.

[26] Zheng K, Xie J (2021). Cluster materials as traceable antibacterial agents. Acc Mater Res.

[27] Higaki T, Li Q, Zhou M, Zhao S, Li Y, Li S, Jin R (2018). Toward the tailoring chemistry of metal nanoclusters for enhancing functionalities. Acc Chem Res.

[28] Wilcoxon JP, Abrams BL (2006). Synthesis, structure and properties of metal nanoclusters. Chem Soc Rev.

[29] Nain A, Tseng YT, Wei SC, Periasamy AP, Huang CC, Tseng FG, Chang HT (2020). Capping 1,3-propanedithiol to boost the antibacterial activity of protein-templated copper nanoclusters. J Hazard Mater.

[30] Setyawati MI, Yuan X, Xie J, Leong DT (2014). The influence of lysosomal stability of silver nanomaterials on their toxicity to human cells. Biomaterials.

[31] Lewinski N, Colvin V, Drezek R (2008). Cytotoxicity of nanoparticles. Small.

[32] Connor EE, Mwamuka J, Gole A, Murphy CJ, Wyatt MD (2005). Gold nanoparticles are taken up by human cells but do not cause acute cytotoxicity. Small.

[33] Pan Y, Neuss S, Leifert A, Fischler M, Wen F, Simon U, Schmid G, Brandau W, Jahnen-Dechent W (2007). Size-dependent cytotoxicity of gold nanoparticles. Small.

[34] Zheng Y, Jiang H, Wang X (2018). Multiple strategies for controlled synthesis of atomically precise alloy nanoclusters. Acta Phys Chim Sin.

[35] Zheng K, Xie J (2020). Composition-dependent antimicrobial ability of full-spectrum Au_x_Ag_25–x_ alloy nanoclusters. ACS Nano.

[36] Zheng K, Setyawati MI, Leong DT, Xie J (2021). Overcoming bacterial physical defenses with molecule-like ultrasmall antimicrobial gold nanoclusters. Bioact Mater.

[37] Tsunoyama H, Sakurai H, Negishi Y, Tsukuda T (2005). Size-specific catalytic activity of polymer-stabilized gold nanoclusters for aerobic alcohol oxidation in water. J Am Chem Soc.

[38] Zheng Y, Liu W, Qin Z, Chen Y, Jiang H, Wang X (2018). Mercaptopyrimidine-conjugated gold nanoclusters as nanoantibiotics for combating multidrug-resistant superbugs. Bioconjugate Chem.

[39] Zheng Y, Wu J, Jiang H, Wang X (2021). Gold nanoclusters for theranostic applications. Coordin Chem Rev.

[40] Zheng K, Setyawati MI, Leong DT, Xie J (2018). Surface ligand chemistry of gold nanoclusters determines their antimicrobial ability. Chem Mater.

[41] Pranantyo D, Liu P, Zhong W, Kang ET, Chan-Park MB (2019). Antimicrobial peptide-reduced gold nanoclusters with charge-reversal moieties for bacterial targeting and imaging. Biomacromolecules.

[42] Xie Y, Liu Y, Yang J, Liu Y, Hu F, Zhu K, Jiang X (2018). Gold nanoclusters for targeting methicillin-resistant *Staphylococcus aureus* in vivo. Angew Chem Int Ed.

[43] Li Y, Zhen J, Tian Q, Shen C, Zhang L, Yang K, Shang L (2020). One step synthesis of positively charged gold nanoclusters as effective antimicrobial nanoagents against multidrug-resistant bacteria and biofilms. J Colloid Interface Sci.

[44] Boda SK, Broda J, Schiefer F, Weber-Heynemann J, Hoss M, Simon U, Basu B, Jahnen-Dechent W (2015). Cytotoxicity of ultrasmall gold nanoparticles on planktonic and biofilm encapsulated gram-positive staphylococci. Small.

[45] Wang Y, Malkmes MJ, Jiang C, Wang P, Zhu L, Zhang H, Zhang Y, Huang H, Jiang L (2021). Antibacterial mechanism and transcriptome analysis of ultra-small gold nanoclusters as an alternative of harmful antibiotics against Gram-negative bacteria. J Hazard Mater.

[46] Landis RF, Li CH, Gupta A, Lee YW, Yazdani M, Ngernyuang N, Altinbasak I, Mansoor S, Khichi MAS, Sanyal A, Rotello VM (2018). Biodegradable nanocomposite antimicrobials for the eradication of multidrug-resistant bacterial biofilms without accumulated resistance. J Am Chem Soc.

[47] Park HJ, Kim JY, Kim J, Lee JH, Hahn JS, Gu MB, Yoon J (2009). Silver-ion-mediated reactive oxygen species generation affecting bactericidal activity. Water Res.

[48] Yang B, Chen Y, Shi J (2019). Reactive oxygen species (ROS)-based nanomedicine. Chem Rev.

[49] Kasuga NC, Yoshikawa R, Sakai Y, Nomiya K (2012). Syntheses, structures, and antimicrobial activities of remarkably light-stable and water-soluble silver complexes with amino acid derivatives, silver(I) *N*-acetylmethioninates. Inorg Chem.

[50] Yuan X, Setyawati MI, Tan AS, Ong CN, Leong DT, Xie J (2013). Highly luminescent silver nanoclusters with tunable emissions: cyclic reduction-decomposition synthesis and antimicrobial properties. NPG Asia Mater.

[51] Haidari H, Kopecki Z, Bright R, Cowin AJ, Garg S, Goswami N, Vasilev K (2020). Ultrasmall AgNP-impregnated biocompatible hydrogel with highly effective biofilm elimination properties. ACS Appl Mater Interfaces.

[52] Xia J, Wang W, Hai X, Shuang E, Shu Y, Wang J (2019). Improvement of antibacterial activity of copper nanoclusters for selective inhibition on the growth of gram-positive bacteria. Chinese Chem Lett.

[53] Nathan C, Cunningham-Bussel A (2013). Beyond oxidative stress: an immunologist’s guide to reactive oxygen species. Nat Rev Immunol.

[54] Memar MY, Ghotaslou R, Samiei M, Adibkia K (2018). Antimicrobial use of reactive oxygen therapy: current insights. Infect Drug Resist.

[55] Yang H, Cai R, Zhang Y, Chen Y, Gu B (2020). Gold nanoclusters as an antibacterial alternative against *Clostridium difficile*. Int J Nanomed.

[56] Zheng K, Setyawati MI, Leong DT, Xie J (2021). Observing antimicrobial process with traceable gold nanoclusters. Nano Res.

[57] Chang TK, Cheng TM, Chu HL, Tan SH, Kuo JC, Hsu PH, Su CY, Chen HM, Lee CM, Kuo TR (2019). Metabolic mechanism investigation of antibacterial active cysteine-conjugated gold nanoclusters in *Escherichia coli*. ACS Sustainable Chem Eng.

[58] Wu Q, Peng R, Gong F, Luo Y, Zhang H, Cui Q (2022). Aqueous synthesis of N-heterocyclic carbene-protected gold nanoclusters with intrinsic antibacterial activity. Colloids Surf A Physicochem Eng Asp.

[59] Tang Z, Liu Y, He M, Bu W (2019). Chemodynamic therapy: tumour microenvironment-mediated Fenton and Fenton-like reactions. Angew Chem Int Ed.

[60] Tang Z, Zhao P, Wang H, Liu Y, Bu W (2021). Biomedicine meets Fenton chemistry. Chem Rev.

[61] Song M, Cheng Y, Tian Y, Chu C, Zhang C, Lu Z, Chen X, Pang X, Liu G (2020). Sonoactivated chemodynamic therapy: A robust ROS generation nanotheranostic eradicates multidrug-resistant bacterial infection. Adv Funct Mater.

[62] Zhao Y, Ye C, Liu W, Chen R, Jiang X (2014). Tuning the composition of AuPt bimetallic nanoparticles for antibacterial application. Angew Chem Int Ed.

[63] Neissa J, Pérez-Arnaiz C, Porto V, Busto N, Borrajo E, Leal JM, López-Quintela MA, García B, Dominguez F (2015). Interaction of silver atomic quantum clusters with living organisms: bactericidal effect of Ag_3_ clusters mediated by disruption of topoisomerase-DNA complexes. Chem Sci..

[64] Meng J, Gao Y, Li W, Wang J, Chen X (2021). Gold nanoclusters exert antibacterial effects against gram-negative bacteria by targeting thiol-redox homeostasis. Talanta.

[65] Gong F, Peng R, Wu Q, Zhang H, Luo Y, Cui Q (2022). Imidazole-stabilized gold nanoclusters with thiol depletion capacity for antibacterial application. Colloids Surf A Physicochem Eng Asp.

[66] Ndugire W, Raviranga NGH, Lao J, Ramström O, Yan M (2022). Gold nanoclusters as nanoantibiotic auranofin analogues. Adv Healthcare Mater.

[67] Buceta D, Busto N, Barone G, Leal JM, Domínguez F, Giovanetti LJ, Requejo FG, García B (2015). López-Quintela López MA. Ag_2_ and Ag_3_ clusters: synthesis, characterization, and interaction with DNA. Angew Chem Int Ed.

[68] Liang J, Xiong H, Wang W, Wen W, Zhang X, Wang S (2018). “Luminescent-off/on” sensing mechanism of antibiotic-capped gold nanoclusters to phosphate-containing metabolites and its antibacterial characteristics. Sens Actuat B Chem.

[69] Kalita S, Kandimalla R, Bhowal AC, Kotoky J, Kundu S (2018). Functionalization of β-lactam antibiotic on lysozyme capped gold nanoclusters retrogress MRSA and its persisters following awakening. Sci Rep.

[70] Li Q, Pan Y, Chen T, Du Y, Ge H, Zhang B, Xie J, Yu H, Zhu M (2018). Design and mechanistic study of a novel gold nanocluster-based drug delivery system. Nanoscale.

[71] Setyawati MI, Kutty RV, Tay CY, Yuan X, Xie J, Leong DT (2014). Novel theranostic DNA nanoscaffolds for the simultaneous detection and killing of *Escherichia coli* and *Staphylococcus aureus*. ACS Appl Mater Interfaces.

[72] Zeng J, Guo Z, Wang Y, Qin Z, Ma Y, Jiang H, Weizmann Y, Wang X (2022). Intelligent bio-assembly imaging-guided platform for real-time bacteria sterilizing and infectious therapy. Nano Res.

[73] Xie Y, Zheng W, Jiang X (2020). Near-infrared light-activated phototherapy by gold nanoclusters for dispersing biofilms. ACS Appl Mater Interfaces.

[74] Hwang GB, Wu G, Shin J, Panariello L, Sebastian V, Karu K, Allan E, Gavriilidis A, Parkin IP (2020). Continuous single-phase synthesis of [Au_25_(Cys)_18_] nanoclusters and their photobactericidal enhancement. ACS Appl Mater Interfaces.

[75] Hwang GB, Huang H, Wu G, Shin J, Kafizas A, Karu K, Toit HD, Alotaibi AM, Mohammad-Hadi L, Allan E, MacRobert AJ, Gavriilidis A, Parkin IP (2020). Photobactericidal activity activated by thiolated gold nanoclusters at low flux levels of white light. Nat Commun.

[76] Nakal-Chidiac A, García O, García-Fernández L, Martín-Saavedra FM, Sánchez-Casanova S, Escudero-Duch C, Román JS, Vilaboa N, Aguilar MR (2020). Chitosan-stabilized silver nanoclusters with luminescent, photothermal and antibacterial properties. Carbohyd Poly.

[77] Nel AE, Mädler L, Velegol D, Xia T, Hoek EM, Somasundaran P, Klaessig F, Castranova V, Thompson M (2009). Understanding biophysicochemical interactions at the nano-bio interface. Nat Mater.

[78] Xie Y, Yang J, Zhang J, Zheng W, Jiang X (2020). Activating the antibacterial effect of 4,6-diamino-2-pyrimidinethio-modified gold nanoparticles by reducing their sizes. Angew Chem Int Ed.

[79] Lin F, Qi Q, Zhang J, Zhou W, Zhang J, Fu P, Zhang X, Qiao X, Liu M, Pang X, Cui Z (2021). From unimolecular template to silver nanocrystal clusters: An effective strategy to balance antibacterial activity and cytotoxicity. ACS Appl Mater Interfaces.

[80] Gilroy KD, Ruditskiy A, Peng HC, Qin D, Xia Y (2016). Bimetallic nanocrystals: syntheses, properties, and applications. Chem Rev.

[81] Kang X, Li Y, Zhu M, Jin R (2020). Atomically precise alloy nanoclusters: syntheses, structures, and properties. Chem Soc Rev.

[82] Zheng Y, Jiang H, Wang X (2018). Multiple strategies for controlled synthesis of atomically precise alloy nanoclusters. Acta Phys Chim Sin.

[83] Zhang Y, Shao Z, Yuan W, Xu H, You X, Liao X (2021). Green and rapid synthesis of cysteine-directed novel AgCu nanocluster hydrogel with good antibacterial activity. Materialia.

[84] Tang Z, Liu S, Chen N, Luo M, Wu J, Zheng Y (2021). Gold nanoclusters treat intracellular bacterial infections: eliminating phagocytic pathogens and regulating cellular immune response. Colloids Surf B Biointerfaces.

[85] Meng J, Hu Z, He M, Wang J, Chen X (2021). Gold nanocluster surface ligand exchange: an oxidative stress amplifier for combating multidrug resistance bacterial infection. J Colloid Interface Sci.

[86] Javani S, Lorca R, Latorre A, Flors C, Cortajarena AL, Somoza Á (2016). Antibacterial activity of DNA-stabilized silver nanoclusters tuned by oligonucleotide sequence. ACS Appl Mater Interfaces.

[87] Yang L, Yao C, Li F, Dong Y, Zhang Z, Yang D (2018). Synthesis of branched DNA scaffolded super-nanoclusters with enhanced antibacterial performance. Small.

[88] Wang L, Li S, Yin J, Yang J, Li Q, Zheng W, Liu S, Jiang X (2020). The density of surface coating can contribute to different antibacterial activities of gold nanoparticles. Nano Lett.

[89] Davies D (2003). Understanding biofilm resistance to antibacterial agents. Nat Rev Drug Discov.

[90] Gupta A, Das R, Tonga GY, Mizuhara T, Rotello VM (2018). Charge-switchable nanozymes for bioorthogonal imaging of biofilm-associated infections. ACS Nano.

[91] Wu J, Li F, Hu X, Lu J, Sun X, Gao J, Ling D (2019). Responsive assembly of silver nanoclusters with a biofilm locally amplified bactericidal effect to enhance treatments against multi-drug-resistant bacterial infections. ACS Cent Sci.

[92] Goswami N, Bright R, Visalakshan RM, Biswas B, Zilm P, Vasilev K (2019). Core-in-cage structure regulated properties of ultra-small gold nanoparticles. Nanoscale Adv.

[93] Wang YW, Tang H, Wu D, Liu D, Liu Y, Cao A, Wang H (2016). Enhanced bactericidal toxicity of silver nanoparticles by the antibiotic gentamicin. Environ Sci Nano.

[94] Zhang J, Chen YP, Miller KP, Ganewatta MS, Bam M, Yan Y, Nagarkatti M, Decho AW, Tang C (2014). Antimicrobial metallopolymers and their bioconjugates with conventional antibiotics against multidrug-resistant bacteria. J Am Chem Soc.

[95] Zheng Y, Liu W, Chen Y, Li C, Jiang H, Wang X (2019). Conjugating gold nanoclusters and antimicrobial peptides: From aggregation-induced emission to antibacterial synergy. J Colloid Interface Sci.

[96] Zheng K, Setyawati MI, Lim TP, Leong DT, Xie J (2016). Antimicrobial cluster bombs: Silver nanoclusters packed with daptomycin. ACS Nano.

[97] Chen W, Chang H, Lu J, Huang Y, Harroun SG, Tseng Y, Li Y, Huang C, Chang H (2015). Self-assembly of antimicrobial peptides on gold nanodots: against multidrug-resistant bacteria and wound-healing application. Adv Funct Mater.

[98] Ye Z, Zhu H, Zhang S, Li J, Wang J, Wang E (2021). Highly efficient nanomedicine from cationic antimicrobial peptide-protected Ag nanoclusters. J Mater Chem B.

[99] Hu W, Younis MR, Zhou Y, Wang C, Xia X (2020). In situ fabrication of ultrasmall gold nanoparticles/2D MOFs hybrid as nanozyme for antibacterial therapy. Small.

[100] Li X, Li S, Bai Q, Sui N, Zhu Z (2020). Gold nanoclusters decorated amine-functionalized graphene oxide nanosheets for capture, oxidative stress, and photothermal destruction of bacteria. Colloids Surf B Biointerfaces.

[101] Zheng K, Li K, Chang T, Xie J, Chen P (2019). Synergistic antimicrobial capability of magnetically oriented graphene oxide conjugated with gold nanoclusters. Adv Funct Mater.

[102] Zheng K, Li S, Jing L, Chen P, Xie J (2020). Synergistic antimicrobial titanium carbide (MXene) conjugated with gold nanoclusters. Adv Healthcare Mater.

[103] Li M, Huang L, Wang X, Song Z, Zhao W, Wang Y, Liu J (2018). Direct generation of Ag nanoclusters on reduced graphene oxide nanosheets for efficient catalysis, antibacteria and photothermal anticancer applications. J Colloid Interface Sci.

[104] Zou X, Zhang L, Wang Z, Luo Y (2016). Mechanisms of the antimicrobial activities of graphene materials. J Am Chem Soc.

[105] Girija AR, Balasubramanian S, Bright R, Cowin AJ, Goswami N, Vasilev K (2019). Ultrasmall gold nanocluster based antibacterial nanoaggregates for infectious wound healing. ChemNanoMat.

[106] Wang X, Wang Z, Fang S, Hou Y, Du X, Xie Y, Xue Q, Zhou X, Yuan X (2021). Injectable Ag nanoclusters-based hydrogel for wound healing via eliminating bacterial infection and promoting tissue regeneration. Chem Eng J.

[107] Liu J, Liu L, Li S, Kang Q, Zhang R, Zhu Z (2021). Self-assembled nanogels of luminescent thiolated silver nanoclusters and chitosan as bactericidal agent and bacterial sensor. Mater Sci Eng C.

[108] Zhu H, Li J, Wang E (2019). Lighting up the gold nanoclusters via host-guest recognition for high-efficiency antibacterial performance and imaging. ACS Appl Mater Interfaces.

[109] Liu X, Cheng Z, Wen H, Zhang S, Chen M, Wang J (2020). Hybrids of upconversion nanoparticles and silver nanoclusters ensure superior bactericidal capability via combined sterilization. ACS Appl Mater Interfaces.

[110] Liu J, Li S, Fang Y, Zhu Z (2019). Boosting antibacterial activity with mesoporous silica nanoparticles supported silver nanoclusters. J Colloid Interface Sci.

[111] Chu G, Zhang C, Liu Y, Cao Z, Wang L, Chen Y, Zhou W, Gao G, Wang K, Cui D (2020). A gold nanocluster constructed mixed-metal metal-organic network film for combating implant-associated infections. ACS Nano.

[112] Xie Y, Zhang M, Zhang W, Liu X, Zheng W, Jiang X (2020). Gold nanoclusters-coated orthodontic devices can inhibit the formation of *Streptococcus mutans* biofilm. ACS Biomater Sci Eng.

[113] Wang L, Hou Q, Zheng W, Jiang X (2021). Fluorescent and antibacterial aminobenzeneboronic acid (ABA)-modified gold nanoclusters for self-monitoring residual dosage and smart wound care. ACS Nano.

[114] Zhuo Y, Zhang Y, Wang B, Cheng S, Yuan R, Liu S, Zhao M, Xu B, Zhang Y, Wang X (2022). Gold nanocluster & indocyanine green based triple-effective therapy for MRSA infected central nervous system. Appl Mater Today.

[115] Wang Y, Cai R, Chen C (2019). The nano-bio interactions of nanomedicines: understanding the biochemical driving forces and redox reactions. Acc Chem Res.

[116] Zheng Y, Wang X, Jiang H (2018). Label-free detection of *Acinetobacter baumannii* through the induced fluorescence quenching of thiolated AuAg nanoclusters. Sensor Actuat B Chem.

[117] Tang H, Li Q, Yan W, Jiang X (2021). Reversing the chirality of surface ligands can improve the biosafety and pharmacokinetics of cationic gold nanoclusters. Angew Chem Int Ed.

[118] Peng Z, Yuan L, XuHong J, Tian H, Zhang Y, Deng J, Qi X (2021). Chiral nanomaterials for tumor therapy: autophagy, apoptosis, and photothermal ablation. J Nanobiotechnol.

[119] Li J, Gao G, Tang X, Yu M, He M, Sun T (2021). Isomeric effect of nano-inhibitors on Aβ_40_ fibrillation at the nano-bio interface. ACS Appl Mater Interfaces.

[120] Marrache S, Dhar S (2012). Engineering of blended nanoparticle platform for delivery of mitochondria-acting therapeutics. Proc Natl Acad Sci USA.

[121] ZhaoY, Zhang Z, Pan Z, Liu Y. Advanced bioactive nanomaterials for biomedical applications. Exploration. 2022;1:20210089. 10.1002/EXP.20210089 PMC10191050 37323697

